# Foraging Patterns of Two Sympatric Wasp Species: The Worldwide Invasive *Polistes dominula* and the Native *Hypodynerus labiatus*

**DOI:** 10.3390/insects17010038

**Published:** 2025-12-27

**Authors:** Sabrina Moreyra, Mariana Lozada

**Affiliations:** Instituto de Investigaciones en Biodiversidad y Medio Ambiente (INIBIOMA), Consejo Nacional de Investigaciones Científicas y Técnicas (CONICET), Universidad Nacional del Comahue (CRUB), Quintral 1250, Bariloche 8400, Argentina; lozadam@comahue-conicet.gob.ar

**Keywords:** foraging strategies, habitat use, interspecific interaction, invasion, vespidae

## Abstract

Invasive insects pose significant ecological challenges due to their interactions with other species, which can have a considerable impact on pre-existent ecosystems. In the present study, we analysed the foraging behaviour of the invasive *Polistes dominula*, which was first detected in NW Patagonia in 2003, and the native wasp *Hypodynerus labiatus*. We evaluated their foraging behaviour in two types of environments: closed habitats with dense vegetation and open habitats without surrounding bushes and trees. Additionally, we recorded the wasps’ feeding choices at three different heights within each context. Our results showed that these sympatric wasps prefer to forage in different environments and in distinct microsite strata within each environment. *Polistes dominula* collected food from the ground level in both open and closed habitats, while *H. labiatus* was more frequently observed in closed areas, gathering resources from higher strata. The observed differences suggest that the collecting sites showed minimal overlap, which may facilitate their coexistence. These findings shed new light on the behavioural processes and interspecific interactions between a highly invasive wasp and a poorly studied native species that inhabit urban and semi urban environments in Patagonia.

## Simple Summary

Invasive insects must overcome important ecological challenges when they arrive in new territories, such as interactions with other species and characteristics of the novel ecosystems. This study analyses the foraging behaviour of two wasps’ species: the invasive *Polistes dominula* and the native *Hypodynerus labiatus*. We compared how these wasps search for food in two types of environments: closed habitats with dense vegetation and open areas with sparse surrounding. We also recorded the heights at which they feed in each type of context. Our findings showed that *P. dominula* preferred foraging on the ground in both habitat types. In contrast, *H. labiatus* mostly foraged higher up in dense vegetation. These differences in habitat use during foraging suggest that both species seem to minimise overlap in their collecting sites, which may facilitate their coexistence. This research improves our understanding of how invasive and native wasps interact in urban and semi-urban areas of Patagonia.

## 1. Introduction

Exotic species must overcome a range of challenges to establish in new territories, including the ecological characteristics of recipient ecosystems and interspecific interactions with both native and other invasive species [1,2,3]. In particular, insect invasions have increased markedly in recent decades, leading to native communities being colonised by multiple exotic species whose interactions can either facilitate or hinder coexistence [4,5]. In line with this, several studies have highlighted the role of behavioural and cognitive plasticity while foraging, as key traits that may favour invasive processes [6,7,8,9]. Additionally, spatial and temporal environmental variability has been identified as key factors facilitating insect invasions, as it can generate niche opportunities that allow exotic species to become established and spread [10]. Given the global rise in insect invasion events [2,4,11], there is a lack of research on novel interactions between native and exotic species. Addressing this gap could enhance our understanding of insect coexistence within dynamic ecosystems.

Among hymenoptera species, social insects such as wasps and hornets represent some of the most abundant invaders worldwide [6,11,12]. The family Vespidae comprises nearly 5000 species with both eusocial and solitary habits, exhibiting complex behaviours that enable them to inhabit diverse ecological contexts [13,14]. The eusocial “paper wasp” *Polistes dominula* (Christ) (Hymenoptera: Vespidae: Polistinae) is a globally invasive species that has become established widely around the world, including Patagonia, Argentina. Although it was first recorded in the region over 20 years ago, little is known about its impact on the invaded areas [15,16,17,18,19]. This generalist wasp hunts arthropods and collects carbohydrate resources, which they take to the nest to feed developing larvae and other adults [20,21]. *Hypodynerus labiatus* (Haliday) is a native potter wasp inhabiting northwestern Patagonia [22] that, despite being first described nearly two centuries ago, remains very poorly studied. This species is solitary and constructs mud nests, each containing a single egg laid by the female together with a variable number of prey—arachnids, lepidopteran larvae, and dipteran larvae—which are consumed by the larva after eclosion [22,23]. Like other wasps, adults of these species primarily consume a variety of carbohydrates, including nectar, pollen, fruit, and sugary substances, as a source of energy [13,23]. This study aims to further investigate the foraging behaviour of these sympatric wasps across diverse disturbed environments, particularly when they gather carbohydrate resources, which constitute one of several food sources used by both species. To this end, we will analyse *P. dominula* and *H. labiatus* foraging responses in both closed areas with dense vegetation and open areas without surrounding bushes or trees. Additionally, we will evaluate wasps’ foraging choices at three different heights within each habitat type.

## 2. Materials and Methods

**Figure 1:**
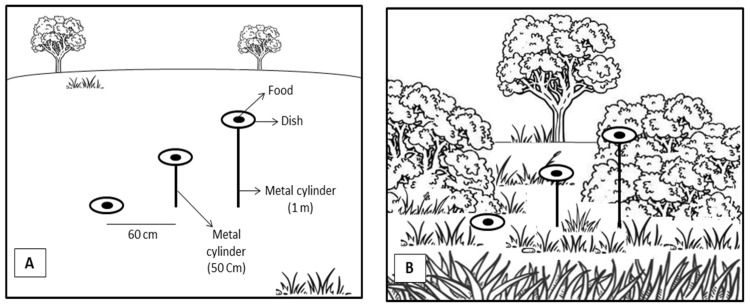
Diagrams of experimental setup in open areas (**A**) and in close environments (**B**).

Field experiments were conducted in urban areas near San Carlos de Bariloche, NW Patagonia, Argentina (41° S, 71° W), in January 2024. We worked in January because both species are abundant and active during this month; in the other months, the native species is usually scarce and harder to find compared with *P. dominula*. All experiments were conducted under similar weather conditions of sunny and calm days. These experiments were performed in macrosites with varying degrees of vegetation, from sparse (without bushes or trees) to densely vegetated areas, representing open and closed environments. Wasps were allowed to collect food from a plastic white dish measuring 7 cm in diameter (feeder), containing a mixture of honey and water solution in a 7:3 ratio (food resource) [9]. We presented three dishes at the same time, each containing the same quality and quantity of resource, located at different heights: ground level, 50 cm, and 1 m above the ground, spaced 60 cm away from each other microsite strata (Figure 1). The dishes were attached with a tape to white metal cylinders of 50 cm and 1 m in height, and were buried in the ground. This setup allowed the wasps to choose among three feeding options at varying heights.

We conducted two experiments to analyse foraging behaviour patterns. In Experiment 1, we evaluated the strata choice of a single *P. dominula* wasp, and in Experiment 2, we assessed the choice of native *H. labiatus* individuals. Both experiments were conducted on the same day and across the same three strata (ground level, 50 cm and 1 m height), in either open or closed environments (treatments), allowing us to compare their foraging behaviour under different vegetation conditions. In Treatment 1, the setup was placed in an open area free of bushes and trees; approximately 4 m in diameter (see Figure 1A). In Treatment 2, the same setup was placed in a closed environment surrounded by dense vegetation, i.e., the dishes were embedded in the vegetation (Figure 1B). The surrounding vegetation consisted of native and invasive species, including grasses, forbs, shrubs, and trees. The most frequent exotic species in the area were *Aegopodium podagraria* “Variegatum”, *Diplotaxis* sp., *Lupinus polyphyllus*, *Tanacetum parthenium*, and *Mentha longifolia*, while the dominant native species were *Fragaria chiloensis*, *Solidago chilensis*, *Alstroemeria aurea*, *Discaria articulata*, and *Maytenus boaria*, among others. We measured vegetation cover at each point, and the dishes were placed in areas with similar cover: approximately 13% at ground level, 9.5% at 50 cm, and 9% at 1 m in open habitats, and 70% at ground level, 55% at 50 cm, and 22% at 1 m in closed habitats.

All experiments were carried out over 25 days, with approximately seven hours of observations per day between 11 AM and 6 PM. In all experiments conducted in open and closed environments, we registered the gathering responses of both species. We recorded which of the three dishes, corresponding to the three strata, the wasps landed on. We began counting landing events after the first stratum was chosen, during a 30 min observation period. This procedure was repeated for each context (Table S1).

In Experiment 1: *P. dominula* was allowed to gather food from the three dishes located at each stratum when the setup was placed in an open area (Treatment 1; *N* = 40) and when it was positioned in a close context (Treatment 2; *N* = 40).

In Experiment 2 the treatments followed the same procedure as in Experiment 1, but analysed the foraging behaviour of *H. labiatus* (*N* = 19).

In each experiment, we recorded the wasp’s strata choice when it was positioned on the feeder with all six legs and began to collect food. After each experiment, the dishes were washed with 96% ethyl alcohol to eliminate potential odour traces (pheromone). The wasp response was recorded by a researcher positioned consistently at a distance of 0.5 m from the setup. It is important to note that only the choice of the first wasp landing on the offered dish was recorded in order to avoid potential social communication mechanisms, which can occur in several eusocial wasp species [24].

### Data Analysis

Given that the data did not follow a normal distribution, the Kruskal–Wallis test was used to compare the wasp’s dish choices at different heights in each context. Pairwise comparisons were conducted using the Mann–Whitney U test. We evaluated the differences in the proportion of wasp species that collected food under different treatments using a contingency table (χ^2^ test). Statistical analyses were performed using IBM SPSS Statistics for Windows (version 23; IBM Corp., Armonk, NY, USA). 

## 3. Results

### 3.1. Experiment 1

**Figure 2:**
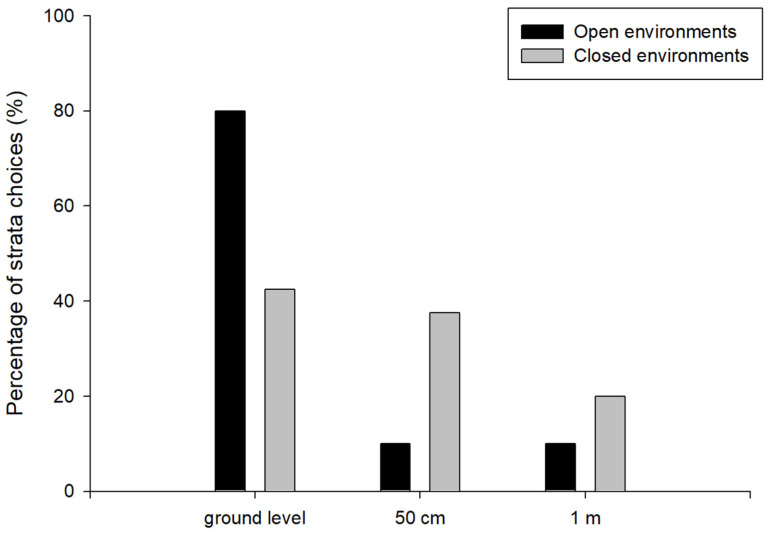
Percentage of strata choices by *Polistes dominula* wasps (dishes placed at different heights: ground level, 50 cm, and 1 m) in open (black bars) and closed (grey bars) environments.

In Treatment 1, when the dishes were placed in an open area, we observed significant differences in the dish choices of *P. dominula* wasps at different heights (χ^2^ = 58.80, df = 2, *n* = 40, *p* < 0.05). Specifically, 32 individual wasps preferred to collect food from the dish placed on the ground, while 4 foragers chose dishes placed at both 50 cm and 1 m heights to collect the resource. Paired comparisons showed significant differences between ground level dishes and those at 50 cm (Z = 6.25, *n* = 40; *p* < 0.05) as well as 1 m (Z = 6.25, *n* = 40; *p* < 0.05) heights. Conversely, no significant differences were found between dishes at 50 cm and 1 m heights (Z = 0, *n* = 40; *p* > 0.05). Of the total number of individuals, 80% of the wasps collected food from the dish placed at ground level, whereas 10% chose the dish at 50 cm and 10% the dish placed at 1 m height (Figure 2).

In Treatment 2, we did not find significant differences in the strata choices of wasps when the setup was located in a close site (χ^2^ = 5.02 df = 2, *n* = 40, *p* > 0.05). The number of wasps that preferred to collect food from the dish placed on the ground was 17, while 15 foragers chose the dish placed at 50 cm height, and 8 individuals chose the dish at 1 m height to collect the resource. No significant differences were found in the wasps’ food collection preferences when analysing pairwise comparisons between the ground and 50 cm (Z = 0.45, *n* = 40; *p* > 0.05), and between 50 cm and 1 m (Z = 1.71, *n* = 40; *p* > 0.05). However, significant differences were found in the wasps’ choice of feeding location between the dish located on the ground and the dish placed at a height of 1 m (Z = 2.15, *n* = 40; *p* < 0.05). In this treatment, the distribution of choices showed that 42.5% of the wasps collected food from the ground-level, 37.5% from the dish placed at 50 cm, and 20% from the dish located at 1 m (Figure 2).

### 3.2. Experiment 2

**Figure 3:**
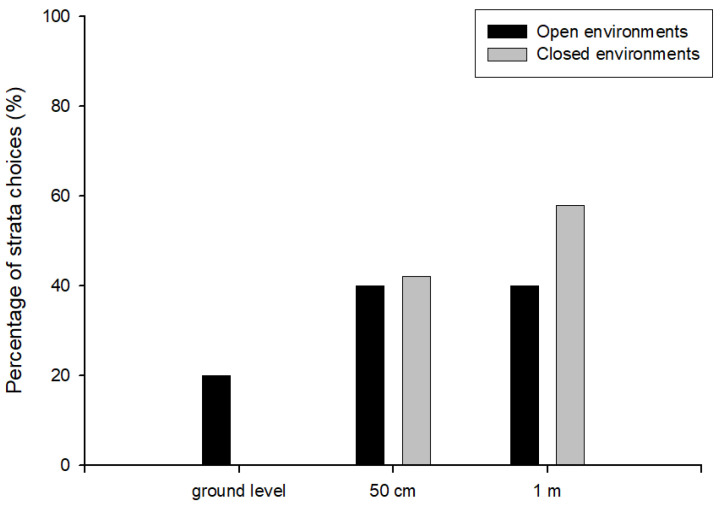
Percentage of strata choices by *Hypodynerus labiatus* wasps (dishes placed at different heights: ground level, 50 cm, and 1 m) in open (black bars) and closed (grey bars) environments.

In this experiment, we analysed the foraging behaviour of *H. labiatus*. When the setup was placed in an open area in Treatment 1, we did not find significant differences in the strata choices made by the wasps at different heights (χ^2^ = 0.44, df = 2, *n* = 19, *p* > 0.05). In this Treatment, only 5 wasps chose to collect food from the dishes. Specifically, out of 19 setups in this environment, only 5 wasps fed from the dishes: 1 forager at ground level, while 2 foragers chose to feed from dishes placed at both 50 cm and 1 m heights. Therefore, in the analysis of paired comparisons, we observed non-significant differences among dishes placed at ground level (Z = 0.59, *n* = 19; *p* > 0.05) compared to dishes at both 50 cm and 1 m heights (Z = 0.59, *n* = 19; *p* > 0.05), as well as between dishes at 50 cm and 1 m heights (Z = 0, *n* = 19; *p* > 0.05). Among the few wasps that collected food from the dishes, 20% did so at ground level, whereas feeding was evenly distributed between the dishes placed at 50 cm and 1 m (40% each) (Figure 3).

Notably, when the setup was positioned in a close area (Treatment 2) we found significant differences in the strata choices made by these wasps at different heights (χ^2^ = 15.31, df = 2, *n* = 19, *p* < 0.05). No individual wasps of *H. labiatus* chose to collect food from the dish located at ground level, while 8 foragers chose the dish placed at 50 cm height, and 11 collected the resource from the dish at 1 m height. We observed significant differences between dishes placed at ground level and those at 50 cm height (Z = 3.14, *n* = 19; *p* < 0.05), as well as between ground level and 1 m height (Z = 3.88, *n* = 19; *p* < 0.05). However, no significant difference was found between dishes at 50 cm and 1 m heights (Z = 0.96, *n* = 19; *p* > 0.05). In this treatment, wasps’ choices were restricted to elevated dishes, with approximately 42% of the wasps collecting food from the dish at 50 cm and 58% from the dish at 1 m, and no visits recorded at ground level (Figure 3).

**Figure 4:**
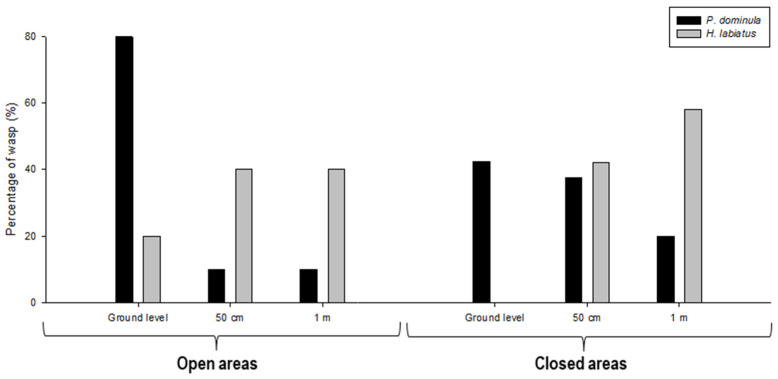
Percentage of wasps collecting food at three heights (ground level, 50 cm, 1 m) in open and closed habitats. Black bars represent *P. dominula* and grey bars represent *H. labiatus*.

We analyse the dish choices located at different heights between wasp species, within each micro-site. In open environments, we found significant differences at ground level (Z = 2.82, *n* = 40, 5; *p* < 0.05) and no significant differences were observed at 50 cm (Z = −1.83, *n* = 40, 5; *p* > 0.05) and at 1 m (Z = −1.83, *n* = 40, 5; *p* > 0.05). In close areas, significant differences were found both for dishes placed on the ground (Z = 3.33, *n* = 40, 19; *p* < 0.05) and at 1 m high (Z = −2.88, *n* = 40, 19; *p* < 0.05), while no significant differences were observed for the feeder located at 50 cm (Z = −0.33, *n* = 40, 19; *p* > 0.05). Moreover, it is important to highlight that we observed differences in the proportion of visits by both wasp species in open and closed micro-sites. When comparing the proportion of visits by both species across each environment type, regardless of dish height, we found few *H. labiatus* individuals (26.5%) visited any dish in open areas, whereas all *P. dominula* individuals did so (χ^2^= 8.18, df = 2, *p* < 0.05). Conversely, in closed habitats, all individuals of both species visited dishes placed within this vegetation context (χ^2^= 13.89, df = 2, *p* < 0.05). When comparing the proportion of wasps’ foraging behaviour at each height within the habitats, we found that both species tend to overlap at 50 cm in closed areas (Figure 4). At all other heights, the species generally exhibited segregation.

## 4. Discussion

This is the first study to demonstrate differential patterns in carbohydrate-gathering behaviour—which wasps use as main energy source— between two sympatric species in urban and semi-urban environments. The observed differences suggest that the collecting sites showed minimal overlap. While *P. dominula* individuals foraged in both open and closed areas, *H. labiatus* preferred to collect food in closed environments. When comparing gathering behaviour at different strata within each environment type, we observed that in open areas, *P. dominula* predominantly collected food at ground level, significantly more than at 50 cm and 1 m. On the other hand, in environments with dense vegetation, foragers gathered resources from the three feeding heights in similar proportions. The few *H. labiatus* individuals that foraged in open areas showed no preference for any strata; in contrast, those wasps collecting resources in closed habitats chose dishes placed at 50 cm and 1 m. In sum, *P. dominula* primarily foraged at ground level in both open and closed habitats, whereas *H. labiatus* was more frequently observed in environments with dense vegetation, collecting resources from elevated feeding sites. Our results revealed that these two species not only tended to forage in different macrohabitats (open versus closed) but also selected different heights within each habitat, suggesting a degree of spatial segregation while foraging in anthropized environments.

The current findings align well with previous research which shows that the long-term coexistence of exotic and native species is influenced by several processes, including spatial and temporal environmental variation and diverse foraging strategies [10,25,26]. Moreover, it has been demonstrated that niche differentiation and resource partitioning play key roles in facilitating the coexistence of invasive and native aquatic insects [27]. Additionally, the sympatric invasive wasps *Vespula germanica* and *V. vulgaris* both gather carbohydrate resources but do so at different microsites or by displaying different foraging behaviours [28]. Furthermore, Wang et al. (2022) [29] found that two aphid species coexist on walnut leaves, occupying specific microhabitats; this niche separation is influenced by physical and behavioural factors during feeding. The results of the present work are in line with the above-mentioned studies, suggesting that differential habitat use patterns may facilitate the coexistence of *P. dominula* and *H. labiatus*, which could potentially mitigate the negative impact of this worldwide invasive wasp on the native potter wasp species. Numerous studies have highlighted the high behavioural plasticity of *P. dominula* across a wide range of invaded environments, a trait that has been linked to its ecological success [16,30,31,32]. This flexibility may also facilitate its coexistence with native species such as *H. labiatus*, particularly in anthropized habitats where resource availability is abundant and variable [33].

It is interesting to note that, since both *P. dominula* and *H. labiatus* act as pollinators of several plant species [21,23], our results suggest that these wasps may play complementary pollinator roles, as they search for and forage on carbohydrate resources at different heights. This pattern has also been observed in other hymenoptera species. For instance, a previous study in another region of Argentina reported that invasive honey bees (*Apis mellifera*) and native social wasps (*Polybia scutellaris*) complement each other in their contribution to plant pollination [34]. The role as pollinators of *P. dominula* and *H. labiatus* should be explored more thoroughly in their ecosystem.

One limitation of this study is that foraging behaviour was evaluated using an experimental design in which only carbohydrate resources were offered. Future studies could explore wasp responses to protein-based food sources. Another limitation is that we only analysed three foraging heights; future studies could examine additional microsite strata and employ other experimental setups. Moreover, it would be interesting to evaluate the foraging behaviour of these two wasps across different seasons, since seasonal variation may influence resource availability, activity patterns, and interspecific interactions. Further studies could assess the foraging behaviour of these species in other biotopes. Likewise, given that previous studies have demonstrated high behavioural plasticity in invasive wasps [8,9,35,36,37,38,39], it seems worthwhile to study and compare the foraging-related cognitive abilities of these two species, as well as those of other sympatric wasps. It would be valuable to further assess whether there are substantial differences in the cognitive flexibility of exotic and native species coexisting in anthropized environments.

## 5. Conclusions

In conclusion, the present research indicates that the foraging patterns of *P. dominula* and *H. labiatus* reflect different gathering strategies and habitat use, which may potentially promote local coexistence. The current findings shed new light on the behavioural processes and interspecific interactions between a highly invasive wasp and a poorly studied native species.

## Data Availability

Data are available on Figshare at 10.6084/m9.figshare.30455936 (accessed on 24 December 2025).

## Supplementary Materials

The following supporting information can be downloaded at: https://www.mdpi.com/article/10.3390/insects17010038/s1, Table S1: Raw data of wasp strata choices under different environmental conditions.
